# Context matters when implementing patient centred rehabilitation models for persons with cognitive impairment: a case study

**DOI:** 10.1186/s12913-021-06206-9

**Published:** 2021-03-06

**Authors:** Katherine S. McGilton, Alexia Cumal, Dana Corsi, Shaen Gingrich, Nancy Zheng, Astrid Escrig-Pinol

**Affiliations:** 1 KITE-Toronto Rehabilitation Institute, University Health Network, Bickle Centre, 550 University Avenue, Suite 236B, Toronto, Ontario M5G 2A2 Canada; 2 Lawrence S. Bloomberg Faculty of Nursing, University of Toronto, 155 College Street, Suite 130, Toronto, Ontario M5T 1P8 Canada; 3 North East Specialized Geriatric Centre, Health Sciences North, 960 D Notre Dame Avenue, Sudbury, Ontario P3A 2T4 Canada; 4 Dalla Lana School of Public Health, University of Toronto, 155 College St., Room 500, Toronto, ON M5T3M7 Canada; 5 The Mar Nursing School (ESIMar), Pompeu Fabra University, Doctor Aiguader, 80, 3a Planta, 08003 Barcelona, Spain

**Keywords:** Cognitive dysfunction, Aged, Rehabilitation centers, Delivery of health care, Focus groups, Health personnel

## Abstract

**Background:**

There is a growing number of older adults with cognitive impairment (CI) that require inpatient rehabilitation, and as such patient centred rehabilitation models have been developed. However, implementing evidence-based models without attending to the fit of the model to the new context could lead to an unsuccessful outcome. Researchers collaborated with administrators and staff in one rural site to adapt a patient centred rehabilitation model of care in the Canadian province of Ontario. This paper reports on the contextual factors that influenced the implementation of the model of care.

**Methods:**

The study takes a case study approach. One rural facility was purposefully selected for its interest in offering rehabilitation to persons with CI. Four focus group discussions were conducted to explore healthcare professionals’ perceptions on the contextual factors that could affect the implementation of the rehabilitation model of care in the facility. Twenty-seven professionals with various backgrounds were purposively sampled using a maximum diversity sampling strategy. A hybrid inductive-deductive approach was used to analyze the data using the Context and Implementation of Complex Interventions (CICI) Framework.

**Results:**

Across the domains of the CICI framework, three domains (political, epidemiological, and geographical) and seven corresponding sub-domains of the context were found to have a major influence on the implementation process. Key elements within the political domain included effective teamwork, facilitation, adequate resources, effective communication strategies, and a vision for change. Within the epidemiological domain, a key element was knowing how to tailor rehabilitation approaches for persons with CI. Infrastructure, an aspect of the geographical domain, focused on the facility’s physical layout that required attention.

**Conclusions:**

The CICI framework was a useful guide to identify key factors within the context that existed and were required to fully support the implementation of the model of care in a new environment. The findings suggest that when implementing a new program of care, strong consideration should be paid to the political, epidemiological, and geographical domains of the context and how they interact and influence one another.

**Supplementary Information:**

The online version contains supplementary material available at 10.1186/s12913-021-06206-9.

## Background

Rehabilitation of persons with cognitive impairment (CI) such as dementia, delirium, or both, is an international priority [1]. With a higher proportion of older adults requiring rehabilitation and the increasing numbers of persons with CI [2], there is a growing concern regarding access to rehabilitation. Approximately 40% of those with hip fractures have a diagnosis of dementia in Canada [3] and globally [4]. For individuals with dementia, they are up to three times more likely to suffer a hip fracture than someone who is cognitively intact [5] Moreover, delirium is the most common complication following hip fracture surgery, affecting 16–44% of patients [6]. Rehabilitation models for persons with CI have emerged following hip fracture surgery that provide evidence of improved functional and health system outcomes as highlighted in a systematic review [7]. Major components of the programs reviewed included daily physical and occupational therapy with various exercise and rehabilitation therapies, with a focus on regaining older adults' strength and functional ability after a hip fracture.  However, this review also identified that most rehabilitation services for individuals who sustain a hip fracture are not designed to meet the complex needs of those who also have CI, such as including approaches that may be needed to engage individuals with CI in rehabilitation [7].

Providing rehabilitative care is critical to the quality of life for older adults post hip fracture as it improves recovery, including those with CI [2]. Evidence also exists that intensive rehabilitation programs for persons with dementia are associated with less risk of long term care admission after discharge from hospital, and is associated with lower risk of mortality than no rehabilitation [2]. However, there are very few rehabilitation facilities that provide care for older adults with CI as there remains an assumption that this population cannot be rehabilitated [8, 9] despite evidence that contradicts this myth [2, 10–12]. Among the patient centred rehabilitation models that exist, there is a lack of specific strategies for implementing these models into other contexts especially when often the majority of the clinicians do not necessarily believe this population can be rehabilitated [8, 13]. Little is known about the factors influencing adaptations of best practice models into a new context.

Researchers from the fields of implementation science and cultural adaptations warn against the dangers of implementing evidence-based models without attending to the fit of the intervention to the context [14]. Context is often referred to as the population being serviced, the different providers who deliver these interventions, and the diversity of the settings [14]. What is known is that the effectiveness of complex interventions is critically influenced by their implementation into a given context [15] and context needs to be considered when analyzing the transferability of strategies [16]. Complex interventions comprise of multiple components, which may act independently or interdependently [17]. Without considering context in this matter, implementation may have varying effects and be dependent on factors that differ between the two systems [16].

However, across the literature, context is often insufficiently defined or omitted all together as reported in a systematic review [18]. Booth et al. describe the Context and Implementation of Complex Interventions Framework (CICI) as a valuable tool for understanding context as it is based on a concept analysis and offers a broad coverage of contextual issues [18]. Pfadenhauer suggested that most frameworks are concerned with evaluating implementation, with a smaller number incorporating an assessment of context [15]. The CICI framework was thus developed to facilitate a structured and comprehensive conceptualization and assessment of context and its' influence on the implementation of complex health interventions. The CICI framework describes seven contextual domains: geographical, epidemiological, socio-cultural, socio-economic, ethical, political, and legal [15].

As the CICI framework is relatively new, only a few researchers have used it to understand implementation challenges. Bardwell et al. used the geographical and legal domains of the CICI framework to understand the outcomes and challenges with implementing supervised consumption service sites in different areas [19], while Evans et al. used the geographical and sociocultural domains as examples of how interventions can be translated across different contexts [16]. Understanding how different contexts influenced implementation is necessary so that empirically tested models can be adapted to fit into new contexts.

For the past 14 years, the lead Principal Investigator (PI) of this current study has focused on addressing issues in rehabilitation of persons with CI and with clinicians developed a patient centred model of care which achieved positive outcomes for persons with CI [10, 20]. The Patient-Centred Rehabilitation Model targeting older persons with CI (PCRM-CI) ([10] was one of the models in the review by Resnick and colleagues [7] which has demonstrated positive outcomes. In addition, health care professionals (HCPs) working with this new population have also embraced their role and have reported satisfaction in their work and rehabiliating persons with CI [21]. The model focuses on four distinct elements: 1) Education and support for clinicians; 2) Patient/Family engagement and education; 3) Strategies for managing the approach to care for someone with CI, including screening for the prevention and treatment of delirium; and 4) Interprofessional rehabilitation care. In 2017, there was an interest to adapt this model of care to a more rural location, based on provincial data that showed that the rehabilitation of persons with CI and hip fracture could be improved in these contexts. For the purposes of this study, a rural area is defined as a town outside of the commuting zones of larger urban centres with a population of typically less than 10,000 people [22].

A rural facility in Ontario was selected as the site for the implementation of the rehabilitation model of care targeting older perons with CI. The facility was a complex continuing care unit which provides continuing, medically complex, and specialized services to older adults over extended periods of time. In the initial implementation stage, we conducted training for the staff including workshops and provided on site part-time facilitators to support implementation of the model of care which is important for the uptake of new practices  [23]. After 6 months of capacity building, focus group discussions (FGDs) were conducted to assess elements of the context that needed strengthening to support the next stages of the adaptation of the model to this rural context.

The purpose of our study was to identify key elements of the context supportive of the next implementation phase of the model of care. These key elements are differentiated into: 1) elements of the context already in place to support the implementation of the model, henceforth referred to as existing elements; and 2) elements of the context that could be introduced at the next  stage to strengthen the chances of successful implementation, henceforth referred to as required elements. By identifying not only elements of the context which needed strengthening, but also those existing elements already in place, we aimed at ensuring that any future system-level changes in the facility would take into account the importance of both these elements for the successful implementation and continuation of this model of care.

## Methods

The study design takes a qualitative case study approach [24, 25] to facilitate an examination of the context in this facility in the initial implementation phase. This approach enabled the exploration of the accounts of multi-disciplinary team members who were involved in the provision of direct care at this facility [26, 27]. The facility was selected by the PI as administrators of the facility had contacted the PI as they wanted to adapt this model of care in order to meet the provincial quality indicators related to the rehabilitation of older adults with hip fracture including those with CI. The site was also  selected because characteristics of the population (patients with and without CI, post hip fracture surgery) at the facility, and system capacity across the region were common to other regions that had low rehabilitation service utilization rates and variations in practice so the lessons learned could be shared across similar regions. By implementing the model, the administrators' goal was to  provide rehabilitation services for this population, in paticular those persons with CI. Purposeful sampling was used which involves choosing information-rich cases to illuminate the research questions [28]. Data was collected through the use of four FGDs.

### Sample and recruitment

We employed a purposive diversity sampling strategy [28] to ensure that the FGDs included participants of varied allied health disciplines and nursing backgrounds. After two focus groups, an appraisal was done to check if all professional categories of the multidisciplinary team were found in the sample. In total, there were 27 HCP participants: 11 Registered Practical Nurses (RPN), four Occupational Therapists (OT), four Physiotherapists (PT), three Rehabilitation Assistants (RA), two Registered Nurses (RN), one Recreation Therapist (RT), one Recreation Therapy Assistant (RTA), one Registered Dietitian (RD), and their demographic information are outlined in Table 1.

**Table 1:** Demographics of focus group discussion participants

Variable	Results (***N*** = 27)
**Age**	
Range	25–47
Mean (±SD)	32.41 (±6.52)
**Sex**	**n (%)**
Male	1 (3.70)
Female	26 (96.30)
**Job title**	**n (%)**
Occupational Therapist	4 (14.81)
Physiotherapist	4 (14.81)
Rehabilitation Assistant	3 (11.11)
Recreation Therapist	1 (3.70)
Recreation Therapy Assistant	1 (3.70)
Registered Dietitian	1 (3.70)
Registered Practical Nurse	11 (40.74)
Registered Nurse	2 (7.40)
**Highest education**	**n (%)**
High school	0 (0)
College	13 (48.15)
University degree	7 (25.93)
Master's degree	6 (22.22)
**Specify master: n (%) from 6 master's degree**	
- Physiotherapy	3 (50)
- Occupational Therapy	3 (50)
**Other**	
- Postgraduate diploma	1 (3.70)
**Previous years & months in rehabilitative care**	
Range	1 month – 23 years
Mean (±SD)	6.86 (±5.93)
**Years & months in current position**	
Range	1 m – 9 years
Mean (±SD)	3.57 (±3.52)

Upon receiving ethics approval from the Research Ethics Board, the PI contacted the facility management and invited them to participate in the research study. Staff were invited to participate in the FGDs via email. The objective of the research was reviewed during the informed consent process. There were no dropouts from the study after informed consent was obtained.

### Data collection

In October of 2018, four focus groups took place in a private room at the facility. Interviews were conducted by an all-female team: a registered nurse with a Doctor of Philosophy (PhD) in nursing and senior scientist with nursing research experience (KM) and a PT (SG) who took notes during the FGDs. The expertise and professional background of the interviewers were shared with participants prior to the focus groups.

The interview guide was developed using the Consolidated Framework for Implementation Research (CFIR) and the topics were chosen based on the framework which focuses on factors that influence the process of implementation [29]. Topics guiding the interview focused on how various contextual factors may influence implementation and included: the influence of the roles of the team members and their interactions; the influence of the physical environment; their interactions with management; the needs of patients with CI; the needs of the staff; their preconceived ideas about rehabilitating persons with CI; economic resources required; norms and rules related to decision making for this population; equity and access to rehabilitation; and concerns around legal issues. The questions were asked by the facilitator without prior testing conducted. Follow up questions to obtain further depth in the participants’ responses were asked by the facilitator. The interview guide developed for this study is provided as Additional File 1.

There were four focus groups ranging from five to ten participants per group, with an average size of eight participants and an average duration of 45 min. Two groups were composed of allied health professionals and two groups were composed of nursing staff. It was not possible to have a mix of interprofessional staff within the focus groups as the staff within these units had diverse workloads. Similarly, we did not aim at having sex balanced groups, rather, the sex ratio of the sample reflects that of the study site, and that of the sector more broadly. Data collectors debriefed after each focus group to discuss new topics, their field notes related to group dynamics, and made revisions to the interview guide as warranted. Data collection was completed when the investigators indicated that data saturation [30] had occurred. No member checking was conducted.

### Data analysis

Data analysis was conducted by a team of five researchers: a male Master’s student in rehabilitation sciences (DW), a female RN student (NZ), a female PhD candidate in Public Health and social scientist (AEP), a female Occupational Therapist with a Master’s degree in Healthcare Quality (DC), and a female Master’s prepared RN (AC). The composition of the research team was mostly female. A similar sex ratio can be observed among HCPs at the study site as well as in the participant sample, which is made up of female HCPs except for 1 male. The FGDs were audio-recorded, anonymized, and transcribed by professionals. NVIVO10 and Microsoft Word 2016 were used to manage data and to conduct analysis.

The analysis was conducted in two consecutive phases following a hybrid inductive-deductive approach [31] used in a previous study by this research team [11]. In Phase 1, we used a thematic, data-driven strategy to coding [32]. Two analysts independently coded each transcript. Then, they met to review the coded transcript, discuss and reach an agreement on discrepancies, and improve the codes. Phase 2 was guided by the CICI framework [15]. When analyzing the data, the CICI framework was more conducive for the analysis as participants highlighted key contextual issues that they felt were in place and identified those that were required for successful implementation to occur. Two analysts examined the thematic codes and categorized them using the CICI framework domains, meeting weekly to review discrepancies. The resulting categorization was then discussed by the full analysis team. The findings below are organized using this categorization guided by the CICI framework domains.

We shared the findings of this study with participants and the study site, and collected their feedback to inform the next stage of the implementation process and future research. We took multiple steps to maintain rigor at several stages of the analysis similar to our previous study [11]. For instance, we accounted for trustworthiness and credibility by: managing data systematically using a project log, incorporating researcher triangulation, establishing a detailed audit trail of documentation, peer debriefing, examining competing explanations, and using reflexivity [33, 34].

## Results

The majority of the data from the FGDs corresponded to three of the seven CICI framework domains: 1) Political, 2) Epidemiological, and 3) Geographical. Within each of these three domains, specific themes surfaced in the FGDs, henceforth referred to as sub-domains of the context. Within each sub-domain, we differentiate between the existing and required elements noted by HCPs. Existing elements refer to components of the context already existing in the facility that would support the implementation of this model, whereas required elements were those identified by HCPs as needed in order to successfully proceed with the implementation. See Table 2 for a summary of the context assessment with the domains, sub-domains, and the key elements identified.

**Table 2:** Existing and required elements within each subdomain of the political, epidemiological, and geographical domains

Domains	Sub-Domains	Key Elements	
1. Political	1.1. Teamwork: Everyone needs to be on the same page	**Existing Elements**	**Required Elements**
		a. Weekly team rounds b. Effective teamwork	c. Role clarity d. Consistent interdisciplinary approach
	1.2. Facilitation: A strategy for change	a. Facilitation through provision of education	b. A dedicated facilitator for implementation
	1.3. Resources: Quality and quantity matter	a. Adequate staffing	b. Staffing complement to match patient care needs c. Equitable access to capacity building opportunities
	1.4. Communication: Effective information sharing to enhance care delivery	a. Written documentation (goal posters, care plans, whiteboards)	b. An increase in face-to-face interactions
	1.5. Preparedness for change: At all levels	a. Positive staff attitude to new initiatives	b. Organizational leadership to create a vision and an action plan at the facility level
2. Epidemiological	2.1. Caring for persons with cognitive impairment: It’s not one size fits all	a. Use of specific models of care b. Family involvement	c. Incorporating routine and familiarity into processes of care d. An individualized care approach
3. Geographical	3.1. Physical layout: Impact of design on care delivery	a. Leveraging the physical environment to incorporate therapy activities	b. Re-designing the unit’s workspace to support staff collaboration

### Political domain

The predominant proportion of the data pertained to the Political domain of the context, which focuses on the distribution of power, assets, and interests within a population, and also includes the health care system and its accessibility with respect to service delivery, leadership, governance, and human resources [15]. Most data within this domain was related to two key contextual aspects within the CICI framework: power and assets. Power relates to the distribution of itself, between those in leadership positions and staff, and also within a team, such as power differences between team members. The distribution of power within a team has an impact on roles, such as how the roles are carried out. Power also relates to how leadership is carried out, especially with regards to communication, and how leadership is needed to overcome barriers to model implementation. Assets relate to access to resources, which could include human resources, such as staffing, or knowledge, examples of which include training or access to training.

The political domain was organized into five sub-domains: 1) Teamwork; 2) Facilitation; 3) Resources; 4) Communication; and 5) Preparedness for Change. These sub-domains and their corresponding *existing* and *required elements* of the context specific to this case study, are described in detail below.

#### Teamwork: everyone needs to be on the same page

Teamwork was identified by participants as a critical component of the political domain, essential for the next stage of the implementation. Participants identified two existing elements already in place at this facility which would facilitate the implementation: the use of team rounds and effective teamwork. One participant observed that the use of rounds was an existing element which allowed the team to monitor patient goals and progress:

We have rounds once a week with the PT/OT, clinical leads, staff, recreational therapist to discuss how the patient’s doing and what goals we need to move forward. It helps. (Focus group (FG) 3, Participant (P) 19, RPN)

Interdisciplinary rounds were noted as useful to enhance communication, resolve discrepancies of approach among team members, and to ensure all team members remain up to date with patients’ care plans and rehabilitation goals.

In addition to team rounds, a participant remarked that good teamwork among nursing staff allowed them to effectively provide care to a patient with responsive behaviours:

We have a patient here that has a lot of behaviours but we all team up to provide care for him and he has his primary RPN, but if that person is on break, we’ll make sure that he’s being monitored closely...and so we take care of each other that way. (FG3, P21, RPN)

This collaboration between members of the nursing team, including the assistance from colleagues when the primary health care provider was not available, was an existing element that supported the implementation of the model of care.

Two required elements to strengthen the context before moving on to the next phase of implementation were identified: the need to understand each team member’s role in patient mobilization and the need for a consistent interdisciplinary approach. The need for role clarity, in particular with respect to who is responsible for the mobilization of patients, emerged as an element that required strengthening for the eventual success of implementing the new model of care:

We need to mobilize people more, people are in wheelchairs, it was very frustrating for me because obviously, that’s what we want, but … we all need to be on board and it’s not just the physio’s job. (FG1, P8, PT)

Although participants commented on having effective teamwork among nursing staff and effective teamwork among allied health staff, there was a divide between the two groups, as inconsistencies remained in terms of how individuals perceived their roles and carried out certain rehabilitation activities. Another required element identified by participants was the need for a person-centred approach involving all HCPs. One participant weighed in on the importance for all team members to embrace a consistent, person-centered team approach:

It all has to do with an interdisciplinary type of approach, it’s not solely one person’s responsibility to be walking a patient to the meal. It’s not the PT role. It’s everybody’s role and it comes back to the whole common goals. And it’s not just the OT’s role to do ADLs [activities of daily living], nursing can do that as well. (FG1, P10, OT)

A consistent interdisciplinary approach requires effective collaboration between nursing and allied health staff, an element that was found to be required in this context in order to proceed with implementation of the model of care. In order for all team members to be on the same page, the continuation of weekly rounds and effective teamwork (existing elements) were deemed important to continue, and role clarification was recommended in order to provide a consistent interdisciplinary approach between groups (required elements).

#### Facilitation: a strategy for change

Facilitation was identified by participants as being critical to bringing about organizational change through the translation of knowledge into practice. One existing element within the organization, and one required element were identified as important factors to support progress towards the next phase of implementation. An existing element included hiring a part time facilitator who participated in developing the workshops for staff, thereby providing education which laid the groundwork for change, however more was required from the facilitator, as outlined by one participant:

It would be great to have [a facilitator] and that would provide us with more knowledge … So we need what follows after that … we get all the information … we need someone like a facilitator so that we can execute [new practices] here. (FG2, P23, RN)

Staff describe that knowledge and education are important, and acknowledged that a part-time facilitator was hired to facilitate educational workshops for this project. However, staff realized that there was also a need for a facilitator to help bring knowledge into practice, to execute the next phase of the implementation process. Thus, a required element identified was the need for a dedicated facilitator to translate knowledge into practical change by providing mentoring at the bedside that was tailored to the uniqueness of their persons with CI. Several participants remarked that this type of facilitator role was required to integrate new knowledge into practice within the local setting:

I think the idea of a facilitator to help us create what we need, using strategies from outside, but adapting it to what we need here. Someone who’s flexible and can help us work through the challenges of what we face in this system. (FG2, P11, OT)

Participants identified the need for education and a  full-time facilitator to be available for the duration of the implementation project to assist with translating newly acquired knowledge into daily practice while ensuring the strategies proposed fit within the local context. 

#### Resources: quality and quantity matter

The availability of resources, including adequate staffing and continued access to knowledge by all staff during all shifts was a recurrent topic in the discussions. One existing element already in place within the organization and two required elements were mentioned as essential to implement this model of care in this context. As an existing element, participants described how the recent addition of a staff member, a physiotherapist, to their team was an important factor to supporting the delivery of quality care, which allowed for a more manageable workload:

It’s always time management … running after it and having to chase after it, and I think things are going to be better now that we have some more staffing here. We have another therapist with us now so workload will be a little bit more manageable. (FG1, P8, PT)

Participants expressed that having an acceptable daily workload would support the upcoming changes at the facility.

Although having an additional staff member was highlighted as an important existing element, participants identified the required need for additional staffing to ensure the success of implementing the model of care. Participants consistently reported on challenges they faced because of inadequate staffing, and the need for more team members, especially in order to make rehabilitation available on evenings and weekends which was currently not in place. When asked about the type of resources required to provide care to patients with CI, participants noted the need for more nursing staff as well as more allied health staff, due to the need to provide rehabilitation consistently throughout their stay based on the needs of the patients:

And I think having more support. Like I think that often right now we’re having no rehab assistance, no rehab OT/PT available on weekends. And evenings. (FG4, P23, RN)

Thus, from the perspective of participants, a staffing complement to match patient care needs would enable the provision of better-quality rehabilitative care. The second required element that emerged from the participant focus groups revolved around the need for access to knowledge for all team members. As one staff member described, while the initital workshops were perceived as useful, many staff were not able to attend because they work part-time, night shift, or weekends only:

… All of us had the opportunity to participate in the training and a lot of nurses did too, but I’m not sure if people who work night shift, people who work part-time and doing weekends they got that training too because many times – majority of times we’re here only seven hours a day working with the patient but the majority of time it’s the nurses who are with the patient they keep rotating. Their shift changes, not like ours which is steady. So, probably that will help if everybody got that training and also that’ll help them feel like part of the team. (FG1, P4, PT)

Providing continuous training to staff from all shifts and disciplines would allow for a consistent knowledge base, thus building capacity and promoting the uptake of the implementation of the rehabilitative model. Thus, ensuring availability of required resources for teams, achieved through adequate staffing and equitable access to knowledge, was recommended to improve the context in preparation for the next stage of implementation.

#### Communication: effective information sharing to enhance care delivery

Communication was recognized as a critical consideration for the implementation of a new model of care. One existing element and one required element emerged as important for implementation preparedness. An existing element involved the use of written documentation, such as goal posters, care plans, and whiteboards. One staff nurse identified the use of care plans to share updates regarding a patient’s status:

Our physio team is usually pretty good with assessing transfers and then they let us know what we’re going to do and they update the care plan. (FG3, P19, RPN)

The use of care plans to record and communicate information was a current approach within the facility that worked well and would be useful  for implementation of the model of care.

To further support the site's preparedness for implementation, one required element identified by participants was the need for in-person communication – beyond email – for use in conveying changes in processes:

People think the easiest way is to communicate through email, but a lot of times it’s not the best way. It’s not – it needs to be explained in person. (FG3, P22, RPN)

Participants observed that general or update emails can be ineffective as they do not promote the asking of clarification questions and thus can limit the use of new resources or uptake of processes that are communicated; an increase in face-to-face communication can facilitate clarification in the form of questions if the need arises. As such, communication is integral to preparing the local context for implementation and would be enabled through the consistent use of both in-person interactions and written documentation.

#### Preparedness for change: at all levels

Preparedness for change is the final sub-domain within the political domain. One existing element and one required element were identified as important means to support the implementation of the rehabilitation model. A positive attitude to new initiatives was recognized:

I think we’re always open to trying new things, … I feel like we’re always evolving here and it’s really good. (FG2, P14, RD)

The staff’s willingness to embrace change was noted as an important factor for the implementation of a new model of care. The need for organizational leadership to create a vision and an action plan at the facility level emerged as a required element to bring about organizational practice change. One participant reflected on how there is a need for a leader to take action:

Somebody needs to say “Okay, you guys over there and you guys over there, let’s meet in the middle”. Because we'll sit and we’ll have these great discussions about things and then reach no conclusion. There’s no action plan, … it’s just let’s discuss all the problems and then leave the room. So lots of great ideas that float around. (FG2, P13, OT)

Leadership to provide a vision for a plan of action and to coordinate how to bring about change would also enable the success of the next implementation phase. Therefore, the staff’s willingness to change and the need for leadership to take action at the facility level were identified as essential for successful program implementation.

### Epidemiological domain

The epidemiological domain, although not as frequently mentioned by participants as the political domain, was also relevant in terms of the context. The epidemiological domain involves the distribution of diseases or conditions, the attributable burden of disease, and the determinants of population needs [15]. The key contextual aspect from this domain was the attributable burden of disease, which includes knowledge related to diseases, including clinical presentation, health management, and care needs.

Within the Epidemiological domain, there is one prominent sub-domain: Caring for Persons with Cognitive Impairment. This sub-domain, including its existing and required elements are detailed in the following section.

#### Caring for persons with cognitive impairment: It’s not one size fits all

An individualized, person-centred approach to care was identified by participants as a central tenet of this sub-domain. Participants highlighted that individuals with CI, specifically delirium and dementia, have unique needs that require a tailored response. Two existing elements and two required elements were identified. Existing elements consisted of the use of specific models of care and family involvement. The use of effective care models for persons with CI was an existing element within the context that supported rehabilitation of this patient population, as highlighted in the following quote:

I’ve done gentle persuasive approach training and I find that’s been pretty useful. I know that that has come out in the last three years or so … that’s helped me work with patients with behaviors and dementia. (FG3, P21, RPN)

In addition, leveraging family involvement in care delivery emerged as an important supporting element existing within this facility:

Especially with dementia because they [the family] can give you tips on what they know works and a little bit of their history … [I] had a few patients that were quite challenging behavior wise and even involving the family to bring in personal belongings and stuff to help the patient feel like it’s more like their home environment helped with some of the behaviors and the anxiety. (FG3, P22, RPN)

Families provide knowledge about persons with CI to HCPs, enabling an individualized approach to care. Using effective care models and including families as partners in care would serve to support the next implementation phase.

In anticipation of meeting the specialized care needs of this population, participants identified two required elements that would further support population-specific, individualized care delivery. For patients with dementia, for example, these included incorporating familiarity and a routine into processes of care, as demonstrated in the following quote:

I feel like that type of patient [with dementia] that we’re focusing on needs routine, needs a familiar face, needs a schedule. (FG1, P2, Rehabilitation Assistant)

The use of routines could assist in the provision of quality care to patients with CI and support the next phase of implementation. Moreover, the need for an individualized approach in patients with CI was identified as a second required element, as highlighted by one OT:

In acute care there’s that focus and everyone has that same level of knowledge, but here those patients will come in and they'll be mixed with everybody else… you have to shift your mindset between patients, that this patient doesn't need what this patient needs, and I feel like a lot of times there’s a bit of a one size fits all kind of approach here. (FG2, P11, OT)

Participants identified that in this facility there needed to be a shift in mindset, from the one-size-fits all approach, to a more individualized approach, in order to provide effective care to patients with CI. Therefore, the use of effective models of care, involvement of family members, incorporating routine and familiarity, and an individualized care approach emerged as important elements to support the provision of care to persons with CI, and to enable the next implementation phase.

### Geographical domain

The third domain that emerged as significant for consideration within the context was the Geographical domain, understood as the broader physical environment, landscapes, and resources, including infrastructure, that are available in a specific setting [15]. Infrastructure was a key aspect of this domain, related to the one sub-domain identified in the analysis, the facility’s physical layout.

#### Physical layout: impact of design on care delivery

The physical layout of the rehabilitation environment was identified as having a significant impact on care delivery and potentially affecting the next phase of the implementation. One existing element and one required element were identified. Participants identified that the location of the dining room, which was part of the physical environment, was leveraged in order to incorporate additional therapy activities during the day:

Everyone does go to the dining room for meals. Breakfast, lunch and dinner they go to the dining room. Ideally with everybody if they are able to ambulate with the required assistance to and from meals as part of their therapy. (FG1, P1, PT)

As the dining room was at a distance for the patients, the design of the unit facilitated patients ambulating more often, which facilitated their recovery.

However, one element of the physical layout required a change as was identified by numerous participants. Within the current geographical context, therapy and nursing were described by HCPs as being siloed from one another, ultimately impacting care delivery. Nursing staff had their station while the allied health care team charted in a different location on another unit. Therefore, the full team did not see each other very often. As such, participants identified that re-designing the unit’s workspace to support staff collaboration would be an important required element for implementation:

So maybe just being more incorporated, being closer to being on the floor, maybe that would just help to influence people, and people, like other staff, would be able to influence us and maybe it would make change smoother. (FG1, P7, OT)

Participants noted that the physical layout reinforced divisions between nurses and allied health professionals. Nursing staff worked in one unit, while the allied health staff worked in an office and rehabilitation gym which were situated on another unit. Of note, participants also described that allied health staff and nursing staff had separate managers, which made the silos even more prominent. Participants perceived that bringing team members closer in proximity to each other would enable more communication and a better working environment which would positively impact on the care provided to patients.  Therefore, maximizing the use of the current physical environment to provide care and making modifications to the layout to bring team members closer together were identified as ways to support the next implementation phase.

## Discussion

Implementing a new model of care implies a change in practice and routines for HCPs. In clinical practice, the context in which HCPs work can facilitate or hinder the staff’s individual responses to these changes in practice. The CICI framework proposed by Pfadenhauer was useful for understanding how HCPs viewed the context 6 months into implementing a patient centred rehabilitation model of care targeting persons with CI [15]. The focus group data mapped onto three key contextual domains of the framework (i.e., political, epidemiological and geographical) and the results demonstrated how they are interconnected and influence one another. The CICI framework was modified to focus on key existing elements within the context which had been supporting the implementation of the model, and those elements that were required to strengthen the context for implementation of the model, which yielded useful considerations for strategies to facilitate practice changes in the future.

Staff perceived that within the political context some key elements existed to enable the adaptation of the model onto their unit, for example effective teamwork, positive staff attitudes to the new initiative, weekly team rounds, and adequate staffing. Indeed, rounding on the unit by members of the interdisciplinary team during the implementation phase of a new program has been shown to provide additional support for implementing a new rehabilitation program [35]. However, the majority of the participants felt that additional changes were required in each of the identified sub-domains to facilitate successful implementation. While team rounds and working on goals for the patients fostered some communication between the allied health staff and nurses, the majority of the staff interviewed noted the presence of a divide between these two groups which influenced their ability to work as an interdisciplinary team necessary to achieve positive patient outcomes [35].

The divide perceived by participants in allied health and nurses has been demonstrated in previous work [36]. In her work Pryor identified that various work practices divide these groups and keep them apart. Allied health staff automatically are seen to have membership on rehabilitation teams whereas nurses have less of a clear role on the team as they have a dual role of management of health conditions and rehabilitation [36]. As a consequence of this divide, opportunities for one to one informal interactions could have been missed which are essential when rehabilitating persons with dementia as the unpredictable nature of their disease requires frequent communication about successful strategies daily. Effective teamwork has been described as key to rehabilitation of older complex adults as team members are required to share and exchange practices that are effective [11, 13, 37].

Participants in this study identified factors that served to perpetuate the divide between the two groups within the political and geographical domains. Political factors influencing this segregation included lack of role clarity between nursing and allied health staff, reporting to different managers, and an unclear vision of the model. Strategies to enhance team cohesion in rehabilitation settings include leaders creating high performing teams [38]. Strategies to this end include recognizing each other’s roles and encouraging individuals to speak up and not be afraid, being open for feedback and creating a sense of safety for staff are suggestions for team cohesion [38]. Moreover, in previous work [11], staff who have implemented best practices related to rehabilitating persons with CI have discussed the need to work outside of their silos and adopt a patient centred approach by being responsive to the needs of all patients which includes nurses walking patients and PTs assisting persons with CI who had responsive behaviors. In addition, leaders within the organization need to determine the desired end state and ensure that the organization is sufficiently aligned to support the change [35, 39], encourage interprofessional team work [40] and have managers set the tone and provide a clear pathway to encourage collaboration and uptake of the new rehabilitation models of care. Furthermore, sustaining new rehabilitation practices includes continuous education, prompting and champions to drive the new practices [40]. Even though a part time resource was hired to facilitate educational workshops, and implement and maintain momentum of this implementation project, the need for full time presence of an external change agent was identified by the participants, and has been highlighted as a required element when implementing best practices in dementia care [41].

In terms of the epidemiological domain, required elements to implement the model included the need to tailor and individualize approaches to care for persons with CI. Previous research has suggested that rehabilitation of persons with CI is achievable and HCPs can learn to modify their practices to achieve positive outcomes; however, it takes creativity, ingenuity, and tailored approaches to rehabilitate persons with CI successfully [11]. These tailoring approaches involve knowing the individual, maintaining routines, and learning the best ways to engage and motivate the person during their rehabilitation journey [11, 12]. Providing feedback to staff and supporting their efforts to tailor their approaches and identifying benchmarks for evaluation success are also essential when implementing new rehabilitation programs [35].

Similar to Bardwell and colleagues [19], the CICI model underscores the importance of considering the geographical domain in order to gain a more robust understanding of the roles these particular external contextual factors may have in shaping model implementation. The segregation between nursing and allied health staff within the political domain was perpetuated by the design flaws within the geographical domain, as nursing staff worked in one unit, while the allied health staff worked in an office and rehabilitation gym which were situated on another unit. Improving the design of the unit to include allied health staff on the rehabilitation unit where nurses were located would also help address group specific use of the built environment. Locating both groups onto one unit would also increase the possibility of sharing successful approaches to tailoring rehabilitation which is essential when rehabilitation persons with CI [11].

In terms of recommendations for implementing rehabilitation models of care into new sites based on the results of this study, the following strategies are outlined: create a vision and action plan; clarify the role of the team members in supporting the program and have the staff report to one manager to ensure accountability; encourage interdisciplinary teamwork by creating opportunities for engagement; include continuous education; provide a full time facilitator to mentor staff until the model is sustained; and provide positive feedback to staff on their attempts to tailor care for persons with CI.

The results of this study advance the field by utilizing the CICI framework to illuminate the importance of considering the political, epidemiological, and geographical domains when adapting a rehabilitation model into a new practice setting and how they interact and influence one another. For implementation studies involving persons with CI, there needs to be a focus on the epidemiological domain, highlighting an individualized approach which takes into account the unpredictable nature of their symptoms and the need for modifications of approaches and strategies to successfully rehabilitate persons with CI. Furthermore, this study underscores the importance of the geographical domain as the physical layout may need to be modified to facilitate and support staff collaboration and sharing of successful strategies to promote improved patient outcomes. Moreover, we modified the CICI framework to incorporate both existing and required elements of the domains, which provides a framework to understand the elements within the context that can be used as a point of reflection of what is going well and what is still required during an implementation project. Future work should involve understanding which elements of the CICI framework should be prioritized when introducing a practice change and to understand how these findings are applicable to other situations.

### Study strengths and limitations

This study provides an in-depth examination of the utilization of the CICI framework to understand the implementation process, however some methodological considerations must be considered when interpreting the findings. We only interviewed HCPs at the point of care and interviews with managers may have revealed different issues related to the context. Interviews with management could have yielded different perspectives and more insights into higher-level systemic issues which needed to be overcome when implementing a new program of care and additional domains of the CICI framework may have become apparent. Strengths of this research include multiple HCPs and the use of a guiding framework to capture the complexity of the implementation process.

## Conclusion

The CICI framework and the results demonstrate how complex implementation of a new model of care is and the importance of context. Numerous models have been developed to assess implementation, but fewer have been created to assess context [15]. The framework was a useful guide to understand which elements existed and which were still required for successful implementation of the model of care.

## Supplementary Information

**Additional file 1.** The interview guide developed for the study.

## Data Availability

The datasets generated and/or analysed during the current study are not publicly available due to possibility of confidentiality being compromised, but are available from the corresponding author on reasonable request.

## Abbreviations

CI: Cognitive Impairment
CICI: Context and Implementation of Complex Interventions (CICI)
FGDs: Focus group discussions
HCPs: Health care professionals
RPN: Registered Practical Nurses
OT: Occupational Therapists
PT: Physiotherapists
RA: Rehabilitation Assistants
RN: Registered Nurses
RT: Recreation Therapist
RTA: Recreation Therapy Assistant
FG: Focus group
P: Participant
