# Supplementary material for: Context matters when implementing patient centred rehabilitation models for persons with cognitive impairment: a case study
Source: BMC Health Serv Res. 2021 Mar 6;21:204. doi: 10.1186/s12913-021-06206-9 (PMC7937255; doi:10.1186/s12913-021-06206-9)
Supplement: Supplementary file 1 — Additional file 1. The interview guide developed for the study. [file 12913_2021_6206_MOESM1_ESM.docx]

# Additional File 1 - The interview guide developed for the study

1. Did you believe that this model of care focused on persons with cognitive impairment changed care practices on your team?

If so, how?

1. At that time, what types of changes did you think the project might result in and how did you think they would (or would not) improve your ability to provide care?
2. What impact on patient care, patients’ experiences of care and their outcomes did you think PCRM might have?
3. Would you please share some specific examples of strategies you’ve employed to rehabilitated persons with CI

2. What do you think is the most rewarding aspect of implementing this type of care within your team?

3. What do you think is the most challenging aspect of implementing this type of care within your team?

4. How would you describe your team’s environment for trying new approaches to patient care?

5. Please tell us what parts of the training content you found most valuable and why?

1. Are there any gaps in the training content? Is there additional training content that you believe would benefit you and your team?
2. What approaches to training (e.g., seminars, bedside instruction, group case-based learning) do you most value/find most effective?
3. Do you feel you have the knowledge and skills you need to provide this type of care to care for older adults post hip fracture with dementia, delirium or both?

- What additional knowledge and/or skills do you believe would be valuable?

1. Do you feel you have the resources and support you need to provide PCRM care?

- What additional resources and/or supports might be beneficial?

6. Have there been changes to the way you and your team work as a team to provide patient care?

1. To what extent is there role clarity? Is there any confusion caused by role overlap?
2. Have there been changes in the way you and your team communicate?
   - If so, please describe these changes
